# Control of Visceral Leishmaniasis in Latin America—A Systematic Review

**DOI:** 10.1371/journal.pntd.0000584

**Published:** 2010-01-19

**Authors:** Gustavo A. S. Romero, Marleen Boelaert

**Affiliations:** 1 Núcleo de Medicina Tropical, Faculdade de Medicina, Universidade de Brasília, Brasília, Distrito Federal, Brazil; 2 Epidemiology and Disease Control Unit, Institute of Tropical Medicine, Antwerp, Belgium; Institut Pasteur de Tunis, Tunisia

## Abstract

**Background:**

While three countries in South Asia decided to eliminate anthroponotic visceral leishmaniasis (VL) by 2015, its control in other regions seems fraught with difficulties. Is there a scope for more effective VL control in the Americas where transmission is zoonotic? We reviewed the evidence on VL control strategies in Latin America—diagnosis, treatment, veterinary interventions, vector control—with respect to entomological and clinical outcomes.

**Methodology/Principal Findings:**

We searched the electronic databases of MEDLINE, LILACS, and the Cochrane Central Register of Controlled Trials, from 1960 to November 2008 and references of selected articles. Intervention trials as well as observational studies that evaluated control strategies of VL in the Americas were included. While the use of rapid diagnostic tests for VL diagnosis seems well established, there is a striking lack of evidence from clinical trials for drug therapy and few well designed intervention studies for control of vectors or canine reservoirs.

**Conclusion:**

Elimination of zoonotic VL in the Americas does not seem a realistic goal at this point given the lack of political commitment, gaps in scientific knowledge, and the weakness of case management and surveillance systems. Research priorities and current strategies should be reviewed with the aim of achieving better VL control.

## Author Summary

Visceral leishmaniasis is a vector-borne disease characterized by fever, spleen and liver enlargement, and low blood cell counts. In the Americas VL is zoonotic, with domestic dogs as main animal reservoirs, and is caused by the intracellular parasite *Leishmania infantum* (syn. *Leishmania chagasi*). Humans acquire the infection through the bite of an infected sand fly. The disease is potentially lethal if untreated. VL is reported from Mexico to Argentina, with recent trends showing a rapid spread in Brazil. Control measures directed against the canine reservoir and insect vectors have been unsuccessful, and early detection and treatment of human cases remains as the most important strategy to reduce case fatality. Well-designed studies evaluating diagnosis, treatment, and prevention/control interventions are scarce. The available scientific evidence reasonably supports the use of rapid diagnostic tests for the diagnosis of human disease. Properly designed randomized controlled trials following good clinical practices are needed to inform drug policy. Routine control strategies against the canine reservoirs and insect vectors are based on weak and conflicting evidence, and vector control strategies and vaccine development should constitute research priorities.

## Introduction

Visceral leishmaniasis (VL) in Latin America is a severe systemic disease caused by an intracellular protozoon, *Leishmania infantum* (syn. *L. chagasi*). VL is a zoonosis: the domestic dog is the main animal reservoir, while foxes and other wild animals play a role in sylvatic transmission [1]–[5]. The parasite is transmitted by a night-biting sandfly, *Lutzomyia longipalpis*, a 2 to 3 mm-long insect well adapted to the peri-domestic environment and distributed throughout Latin America [6]–[10]. *L. infantum* is also transmitted by *Lu. cruzi* in Brazil [11] and *Lu. evansi* in Colombia, and Venezuela [12],[13]. Clinically, VL is characterized by prolonged fever, weight loss, hepatomegaly, splenomegaly, hypergammaglobulinemia and pancytopenia and it is usually fatal if not adequately treated [14]. Not all *L. infantum* infections lead to overt clinical disease: in Brazil ratios of 8–18 incident asymptomatic infections to 1 incident clinical case were described [15]–[17]. Risk factors for the development of clinical disease are only partially understood. Some studies suggest that the susceptibility to VL could be genetically determined [18]–[22]. Malnutrition places children at higher risk [23],[24]. Other studies identified being a young male and the presence of animals in the neighborhood [25], living in houses with a inadequate sewage system and waste collection [26], and residence in an urban slum or in areas with green vegetation [27] as risk factors.

The VL disease burden in Latin America is not exactly known because most countries lack effective surveillance systems [28]–[30]. Brazil declared a total of 50,060 clinical VL cases between 1990 and 2006 and this number accounts for 90% of all reported VL cases in the Americas, but is subject to substantial underreporting [29],[31]. The country reported so far 176 HIV-coinfected VL cases [32] but has a significant number of asymptomatic co-infected individuals [33],[34]. Whereas VL was initially concentrated in the poor rural areas in the northeast of the country, since the 1980s epidemics have occurred in major cities such as Belo Horizonte, Campo Grande, Natal, and others [35]–[37]. Some of these urban VL outbreaks were attributed to the migration of families from the rural areas to the peri-urban slums after periods of prolonged drought. Whereas the reported VL incidence in the 1980s averaged at 1,500 cases per year, this figure increased to an average 3,362 per year between 2000 and 2006 [31]. The disease has gradually spread south and eastward and is reported since 1999 from the states of São Paulo and Mato Grosso do Sul [38]. Human VL cases have also been reported from Honduras [39], Venezuela [40], Paraguay [41] and Argentina [42]. Sporadic and/or import human or canine cases were described in Chile [43], Ecuador [44], Bolivia [45], Mexico [46], Costa Rica [47], and French Guyana [48]. A geographically referenced database providing links to published literature about the spatial distribution of VL can be accessed on http://apps.who.int/tools/geoserver/www/ecomp/index.html (Accessed on September 19 2009).

Control of VL in the Americas has proved challenging. Early diagnosis and treatment is essential for the patient, but has limited impact on transmission if the main animal reservoir or insect vectors are not tackled [49]. Some studies showed a decreased incidence of VL in both dogs and children following serological screening and culling of seropositive dogs [50],[51], but this control strategy is increasingly debated [52]. Human VL incidence remained high in Brazil despite intensive application of this strategy in recent years [31]. Lack of impact has been attributed to the low sensitivity of the diagnostic tests, the long delay between diagnosis and culling and the low acceptance of culling by dog owners. Mathematical modeling suggests that vector control and vaccination of dogs would be more efficacious than dog culling [49]. Treatment of infected dogs is not an effective strategy as relapses are frequent, and dogs quickly become infectious again [53]. A controlled trial in a different setting of zoonotic VL (Iran) showed how the use of deltamethrin-treated dog collars reduced the risk of infection in dogs (by 54%) and in children (by 43%) [54]. Another controlled trial in Brazil showed only a modest effect on canine seroconversion rates [55] in spite of the proven effect of deltamethrin-impregnated dog collars on vector density [56].

In the Mediterranean region, where VL is also zoonotic with dogs playing a role as main reservoirs, human cases and canine cases are treated with antiparasitic drugs. In Europe, individual measures to protect dogs from sand fly bites using insecticides are common practices, but no public health surveillance and control interventions such as those applied in Brazil are in place [57].

Recently, the governments of India, Bangladesh and Nepal launched a VL elimination initiative, aiming to reduce the annual incidence of VL to less than 1/10,000 population by 2015 [58]. The strategy exploits recent technological developments in diagnosis, drugs and vector control [59]. Though the transmission pattern in this region is totally different, with *L. donovani* being the causative agent, a different sandfly vector (*P. argentipes*) and -most importantly- anthroponotic instead of zoonotic transmission, we wanted to examine whether there is a scope for VL elimination or at least improved control in the Americas. Given the heterogeneity in causative species, vector and transmission pattern, evidence on VL control tools from one region cannot be readily extrapolated to another. We report a review of the literature on the effectiveness of novel VL control tools and strategies in Latin-America structured around diagnosis of human and canine VL, treatment of human cases and control of the animal reservatoir and arthropod vectors.

## Methods

The review on VL control interventions was structured around the following topics: (i) Diagnosis of human VL; (ii) Treatment of human VL; (iii) Diagnosis of canine VL; (iv) Control of the animal reservoir and vector. Box 1 shows the Medical Subject Heading (MeSH) terms and keywords used in the search per topic. We searched for English, Portuguese and Spanish–language articles in MEDLINE, LILACS, as well as the Cochrane Central Register of Controlled Trials from 1960 to November 2008. We considered only original research, mainly but not exclusively intervention trials, diagnostic accuracy studies and observational studies, with scope targeted to American VL. Additional articles were obtained through citation tracking of review and original articles.

**Box 1. Keywords and MESH Headings Used for Literature Searches**

**Diagnosis of human VL:** For the PubMed search: **(visceral leishmaniasis OR kala-azar OR** ***L.infantum OR L. chagasi OR L.donovani OR Leishmania infantum OR Leishmania chagasi OR Leishmania donovani*** **) AND (diagnostic accuracy OR diagnostic performance OR sensitivity OR specificity OR validation) AND “Americas” [MeSH].** For the LILACS search the key-words: **leishmaniasis AND visceral AND (diagnosis OR DAT OR dipstick)** were used.

**Treatment of human VL:** For the PubMed search the following key-words were used: (**visceral leishmaniasis OR kala azar OR** ***L. chagasi*** **OR** ***L donovani*** **) AND (amphotericin b OR glucantime OR sodium stibogluconate OR miltefosine OR sitamaquine** **OR pentavalent antimonials OR paromomycin) AND “Americas” [MeSH]**. For the LILACS search the key-words: **leishmaniasis AND visceral AND treatment** were used. For the *Cochrane Central Register of Controlled Trials* we used the term **visceral leishmaniasis** because the search with the key-words and MeSH terms used for the PubMed searching failed to retrieve any paper.

**Diagnosis of canine VL:** For the PubMed search: **(canine visceral leishmaniasis OR** ***L.infantum OR L.chagasi OR L.donovani OR Leishmania infantum OR Leishmania chagasi OR Leishmania donovani*** **) AND (diagnostic accuracy OR diagnostic performance OR sensitivity OR specificity OR validation) AND “Americas” [MeSH].** For the LILACS search the key-words: **canine** AND **leishmaniasis AND visceral AND diagnosis** were used.

**Control of the animal reservoir and arthropod vector:** for the PubMed search: (**visceral leishmaniasis OR** ***Leishmania chagasi*** **OR** ***L chagasi*** **OR Kala-azar OR** ***Leishmania infantum*** **) AND “Americas” [MeSH] AND control.** The LILACS search was performed using the term **visceral leishmaniasis OR leishmaniose visceral OR leishmaniasis visceral** because of the failure to retrieve any paper when using the PubMed approach.

In a next step, the titles, abstracts and if necessary the full text of the studies was examined to identify relevant papers for the review. Data were extracted by one researcher directly from the full length articles to structured tables containing all the descriptive variables and relevant outcomes. The inclusion criteria, data extracted for each item and summary measures are listed below.

### Human diagnosis

As we have stated above, we set out to examine whether the existing control tools allow for elimination of VL in the Americas. The goal of elimination requires diagnostic and therapeutic tools that are very easy to use and can be easily decentralized. The World Health Organization now considers two ‘rapid diagnostic tests’ as appropriate for the diagnosis of VL in control programs: the Direct Agglutination Test (DAT) based on whole promastigotes of *L. donovani* or *L. infantum* and the rK39-ICT [60]–[62]. As it was not our intention to go into a full review of the available diagnostic tools for VL, we have excluded PCR and serological tests that require substantial laboratory equipment, even though there is extensive experience with the use of IFAT and ELISA tests in the Americas. Moreover, the clinical benefit of antigen-detection and PCR tests still needs to be demonstrated [63],[64]. We therefore limited our systematic review to DAT and rK39-ICT.

The eligibility criteria included: original studies evaluating the DAT or the rK39 immunochromatographic test (ICT); clinical visceral leishmaniasis diseases in humans as target condition; adequate reference classification; absolute numbers of true-positive, true-negative, false-positive and false-negative observations available or derivable from the data presented. Accuracy measures were summarized as sensitivity and specificity.

### Human treatment

Clinical trials including uncontrolled and retrospective studies with description of the following characteristics: intervention; case definition; follow-up schedule; therapeutic endpoints; control group; and efficacy measure defined through cure and failure proportions for each treatment.

### Canine diagnosis

Original studies evaluating any diagnostic test for canine leishmaniasis; *Leishmania* infection and/or VL disease in domestic dogs as target condition; adequate reference classification; absolute numbers of true-positive, true-negative, false-positive and false-negative observations available or derivable from the data presented. Accuracy measures were summarized as sensitivity and specificity.

### Vector control and animal reservoir control

Field trials of control measures (canine culling, impregnated dog collars, canine vaccination, insecticide spraying, insecticide treated bednets, environmental management) evaluating at least one control measure; description of the intervention under analysis; target population, sampling and randomization process; adequate case definitions for asymptomatic infection or VL; definition of outcomes related to humans, dogs or sand flies; at least one effect measure; and at least one point estimation for the magnitude of the expected effect.

## Results

### Human diagnosis

**Figure 1:**
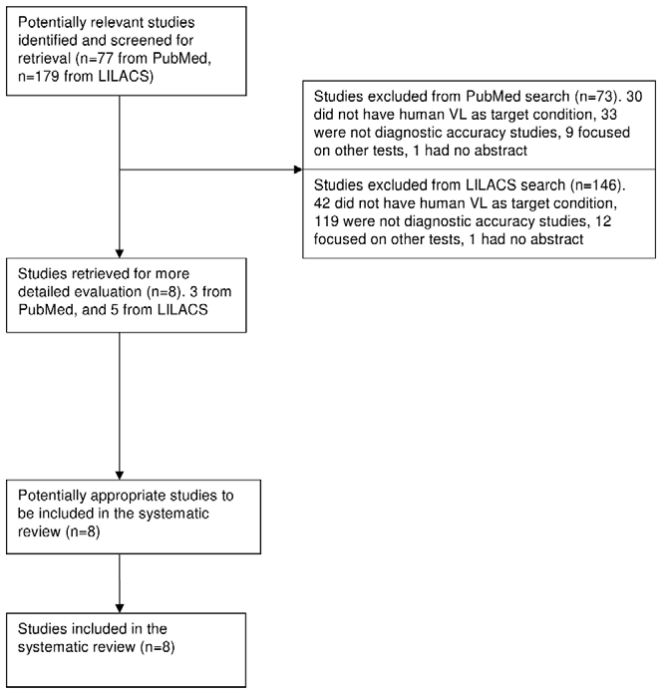
Flow of inclusion of studies on human VL diagnosis.

A Medline search generated 77 papers, and LILACS 179. After screening the titles and abstracts of those papers for evaluations of the DAT or rK39 in human VL, we retrieved eight original papers (Figure 1 and Table 1). We report only descriptive statistics of sensitivity and specificity estimates; without drawing conclusions about differences in these parameters between tests and discuss them in comparison with results of a meta-analysis by Chappuis et al. [65].

**Table 1:** Main characteristics of diagnostic accuracy studies reporting on tests for human visceral leishmaniasis in Latin America.

Country	Type of study	Diagnostic test	Reference test	Number of confirmed VL	Sensitivity	Number of controls	Specificity	Ref.
Brazil	Phase-2	DAT	Not described	33	94%	173 OD***	100%	[66]
						178 HEC****	100%	
Brazil	Phase-2	DAT	Parasitology or improving after antimonial treatment	16	100%	102 OD	100%	[67]
						105 HEC	100%	
Brazil/other	Phase-2	FD-DAT*	Parasitology	36	100%	42 OD	100%	[68]
						19 HEC	100%	
Venezuela	Phase-2	FD-DAT	Parasitology	30	100%	20 OD	100%	[69]
						19 HEC	100%	
Brazil	Phase-2	FD-DAT	Parasitology	88	96.6%	85 OD	97.6%	[70]
						20 HEC	100%	
Venezuela	Phase-2	rK39 ICT	Composite reference **	41	87.8%	76 OD	100%	[71]
Brazil/other	Phase-2	rK39 ICT	Parasitology	36	85.7%	42 OD	80.9%	[68]
						19 HEC	84.2%	
Brazil	Phase-2	rK39 ICT	Parasitology	128	90%	50 OD	100%	[72]
						10 HEC	100%	
Brazil	Phase-3	rK39 ICT	Parasitology	213	93%	119 OD	97%	[73]

*FD-DAT: Freeze-dried DAT.

**Composite reference: at least 2 positive tests out of 4 (bone marrow, IFAT, CIEP, Western blot).

***OD : patients with other, potentially cross-reacting infectious diseases.

****HEC: Healthy Endemic Controls.

Phase 2: Case-Control design, laboratory based study on banked serum samples.

Phase 3: Prospective clinical study, recruiting representative patients, all presenting with febrile splenomegaly.

#### I: Direct agglutination test (DAT) for VL

Andrade et al (1989) were the first to report a proof-of-principle evaluation of the DAT in Brazil [66]. A recent meta-analysis of the DAT performance showed sensitivity and specificity estimates of 94.8% (95%CI: 92.7–96.4) and 97.1% (95%CI: 93.9–98.7), respectively [65]. The performance of DAT was neither influenced by the region nor by the *Leishmania* species. However, this meta-analysis included only two studies from Latin –America, both from Brazil, and both with small sample sizes. Garcez et al (1996) reported 100% sensitivity on 16 parasitologically confirmed VL cases and 98.3% specificity on a mixed group of 65 healthy endemic controls and patients with other diseases [67]. Schallig et al (2002) reported 100% sensitivity on 21 confirmed VL cases and 100% specificity on 19 healthy controls and 42 samples of patients with other diseases [68]. More recently, Teran-Angel et al (2007) reported 100% sensitivity on 30 confirmed VL patients in Venezuela and 100% specificity on 39 controls [69]. Pedras et al (2008) compared the freeze-dried DAT (FD-DAT) and a locally produced DAT with 3 other serological tests (rK39 ELISA, ELISA-*L. chagasi* and IgG-IFAT) and concluded that the FD-DAT was the most efficient, with 96.6% sensitivity (n = 88) and 98.1% specificity (n = 105) [70]. All reported studies are laboratory-based, no large prospective clinical studies evaluating the DAT have been reported from the Americas.

#### II: rK39-based immunochromatographic test (ICT)

Delgado et al (2001) evaluated the rK39-ICT in Venezuela, reporting 87.8% sensitivity (36/41 confirmed VL) and a specificity of 100%. The lower sensitivity was attributed to the fact that the false negative sera had been kept at −70° for more than 10 years [71]. A meta-analysis of 13 studies of the rK3 ICT by Chappuis et al (2006) showed sensitivity and specificity estimates of 93.9% (95%CI: 87.7–97.1) and 95.3% (95%CI: 88.8–98.1), respectively, with some regional variation [65]. This meta-analysis included only two studies from Latin-America [68],[72]. De Assis et al (2008) confirmed the excellent diagnostic performance of rK39-ICT in a prospective study in Brazil, with 93% sensitivity on 213 confirmed VL cases and 97% specificity on 119 controls with clinical suspicion of VL but with confirmation of other diseases [73]. On this basis, it seems that rK39 based diagnosis can be adopted in clinical practice, though each new brand put on the market should be evaluated in proper phase-3 designs.

#### III: Key questions for control

1. What should be the diagnostic algorithms for VL for use in primary health care and in active case detection campaigns?
2. How to assure the quality of available VL rapid diagnostic tests?
3. How to define asymptomatic infected individuals (and how to manage them?)
4. How to improve clinician's awareness about the possibility of *Leishmania* co-infection in HIV/AIDS cases?

#### IV: Questions for research

1. What can be the contribution of novel (molecular) parasite detection tests to clinical diagnosis?
2. What is the performance of diagnostic assays in HIV-*Leishmania* co-infections?
3. What is the performance of antibody-assays in patients from areas with sympatric circulation of parasites causing cutaneous leishmaniasis?

### Canine diagnosis

**Figure 2:**
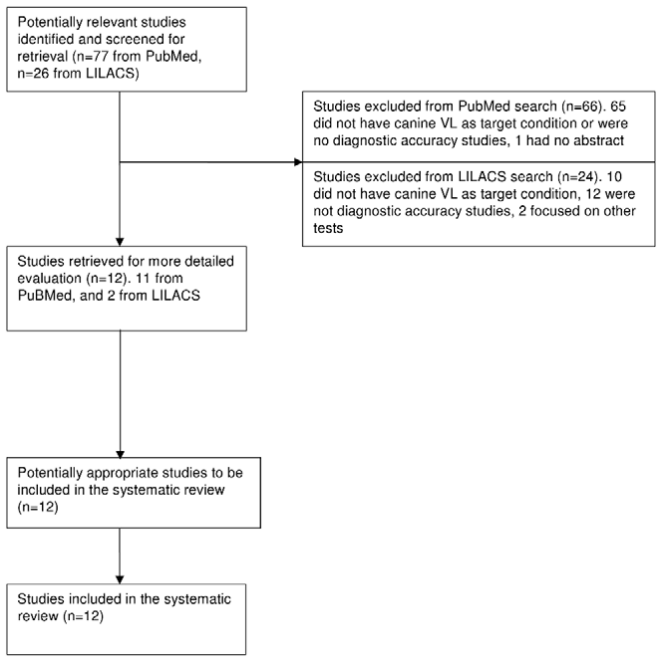
Flow of inclusion of studies on canine VL diagnosis.

Seventy-seven papers were retrieved from Medline/PubMed search and 11 of them were considered relevant. The LILACS database search retrieved 26 papers of which 2 were considered relevant, but 1 was already obtained from the PubMed database (Figure 2). Finally, 12 papers were included in the review, covering 5 serological tests for canine VL: IFAT, ELISA, dot-ELISA, DAT, and rK39-ICT [66], [69], [74]–[83]. IFAT has been the test adopted by the Brazilian Ministry of Health for its dog screening-and-culling campaigns. Published estimates for sensitivity range from 72–100%, for specificity 52–100% (Table 2). The moderate sensitivity and specificity of this test, the long turn-around time between sample taking and culling, and the complexity of its execution have been invoked as one of the reasons for the low effectiveness of the culling campaign. Several ELISA tests have been evaluated, with assays based on homologous antigens usually showing higher sensitivity. Evans et al (1999) showed a higher sensitivity of ELISA compared to IFAT and pleaded for a revision of the screening policy [84].

**Table 2:** Main characteristics of diagnostic tests for canine visceral leishmaniasis in Latin America.

Country	Diagnostic test	Reference test	Number of confirmed VL	Sensitivity	Number and type of controls	Specificity	Ref.
Brazil	IFAT	Parasitology	46	78%	102 NEC	100%	[74]
Brazil	IFAT	Parasitology	21	100%	14 NEC	100%	[66]
Brazil	IFAT	Parasitology	112	72%	20 NEC	100%	[75]
					20 OD	52%	
Brazil	IFAT	CRS	36	100%	67 EC	66%	[76]
Brazil	cELISA	Parasitology	46	98%	102 NEC	99%	[74]
Brazil	cELISA	Parasitology	21	71%	14 NEC	86%	[66]
Brazil	cELISA	Parasitology	106	98–100	25 HEC	100%	[77]
Brazil	cELISA	Parasitology	112	95%	20 NEC	100%	[75]
					20 OD	64%	
Brazil	cELISA	Parasitology	76	95%	33 NEC	100%	[78]
Brazil	cELISA	Parasitology	50 symptomatics	88%	25 NEC	100%	[79]
			50 asymptomatics	30%	14 OD	64%	
Brazil	rK39 ELISA	Parasitology	106	98.1%	25 HEC	100%	[77]
Brazil	rK39ELISA	Parasitology	50 symptomatics	100%	25 NEC	100%	[79]
			50 asymptomatics	66%	14 OD	71%	
Brazil	rK26 ELISA	Parasitology	106	99.1%	25 HEC	100%	[77]
Brazil	rK26ELISA	Parasitology	50 symptomatics	94%	25 NEC	100%	[79]
			50 asymptomatics	66%	14 OD	57%	
Brazil	rA2ELISA	Parasitology	50 symptomatics	70%	25 NEC	100%	[79]
			50 asymptomatics	88%	14 OD	93%	
Brazil	Dot-ELISA	Parasitology	37	97%	63 HEC	100%	[80]
					30 NEC	100%	
Brazil	DAT	Parasitology	21	71%	14 NEC	71%	[66]
Brazil	DAT	Parasitology	112	93%	20 NEC	100%	[75]
					20 OD	95%	
Brazil	FD-DAT	CRS*	36	100%	67 EC	89.5%	[76]
Venezuela	FD-DAT	Parasitology	26	85%	16 HEC	100%	[69]
Brazil	rK39 ICT	CRS**	74	72	101 HEC	61%	[81]
Brazil	rK39 ICT (6 formats)	Clinical + IFAT	50	84–96%	50 HEC	100%	[82]
					14 OD	100%	
Brazil	rK39 ICT	Parasitology	76	83%	33 NEC	100%	[78]
					25 OD	84%	

DAT : variable cut-offs were used, and different antigens, see original papers.

cELISA: ELISA based on crude soluble antigen; rELISA: ELISA based on recombinant antigens; FD-DAT: Freeze-dried DAT.

*CRS: Composite Reference Standard: positive if direct microscopy or culture or PCR positive.

**CRS: Composite Reference Standard:Positive if ELISA or PCR positive.

NEC: healthy dogs from non-endemic areas.

OD: dogs with other, potentially cross-reacting infectious diseases.

HEC: healthy dogs from endemic areas.

Recently more “user-friendly” diagnostics as the DAT and a canine version of the rK39-ICT were evaluated with good results. For the freeze-dried DAT sensitivity ranged from 85–100%, specificity 89–100% [65],[76],[78] and for the rk39-ICT sensitivity ranged from 72–96%, specificity 62–100% [81],[82]. The main advantage of these rapid tests would be to shorten the delay between diagnosis and culling/treatment. However, the reported estimates of sensitivity in the above studies depend on the type of dogs included in the “true cases” group with higher sensitivity observed in symptomatic than in asymptomatically dogs, and unfortunately, several evaluations failed to include an adequate sample of asymptomatically infected dogs. The sensitivity of the test in asymptomatic dogs is crucial for a control strategy, as those dogs are infectious, and should be targeted by the campaign.

Sensitive antigen detection tests as PCR might become a relevant marker of infection in the future with the advantage that they can still be used in vaccinated dogs that will be serologically positive because of the vaccine. However, Quinnell et al (2001) showed in a longitudinal study of naturally infected dogs how the sensitivity of PCR was high early after infection but declined to 50% thereafter. The sensitivity of serology also varied with time, being lowest at the time of infection but clearly superior thereafter (93–100%). They concluded that PCR was most useful for detection of active disease, and considered serology as more adequate for the detection of infection [84].

#### I: Key questions for control

1. What is the most cost-effective diagnostic strategy for a screen-and-treat or screen-and-cull campaign? Novel screening strategies based on combined, parallel or sequential use of current available tests need to be validated.

#### II: Questions for research

1. How to distinguish an antibody response due to natural infection from that produced after vaccination in dogs?
2. What can be the contribution of novel, molecular, parasite detection tests to clinical diagnosis in dogs?
3. What is the value of the current diagnostic tests in terms of dog infectivity for sandflies?

### Human treatment

Thirty-nine papers were retrieved from Medline/PubMed search and four of them were considered relevant. The LILACS database search retrieved 42 papers of which 24 were not available from the PubMed database. Three of those 24 studies were considered relevant, one of them, was previously identified through the PubMed search. One paper was identified through specific author's name searching in PubMed. The Cochrane Central Register of Controlled Trials search retrieved 103 trials, three of them were conducted in the Americas but all were also identified through the PubMed and LILACS searches. Finally, seven papers were included for review [85]–[91].

**Figure 3:**
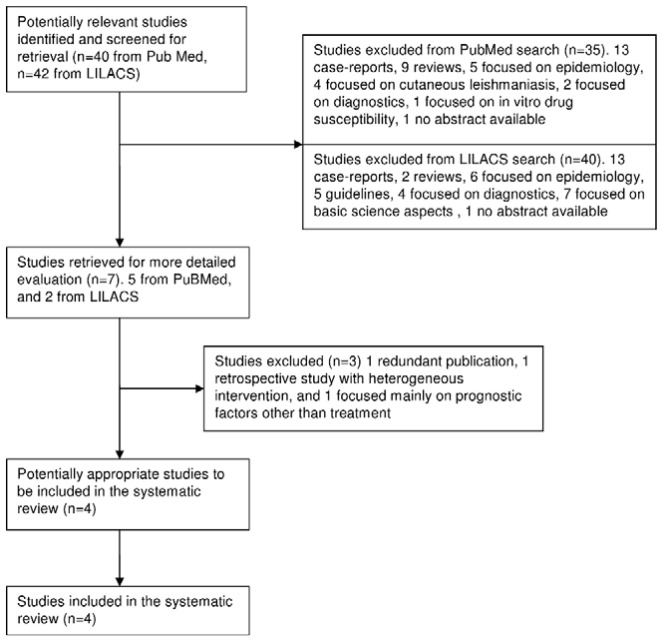
Flow of inclusion of studies on VL treatment.

Three papers were excluded from further analysis, one because it was a second publication on the same trial [88], one for being a retrospective study with heterogeneous therapeutic interventions with meglumine antimoniate and case definition based on clinical findings plus positive serology without description of the methods and test cut-off. A minority of cases was diagnosed through parasite identification [85], and one paper because it was a case-control study focusing on prognostic factors [87]. The flow for the selection and a summary of the reviewed studies appears in Figure 3 and Table 3.

**Table 3:** Main characteristics of selected studies reporting treatment of human visceral leishmaniasis in Latin America.

Country	Type of study	Number of subjects	Mean patient age (years)	Treatment interventions	Dose and route	Follow-up period	Outcomes (%)	Ref.
Brazil	Open-label	10	20.0	Amphotericin B cholesterol dispersion	2.0mg/kg/d for 10 d. I.V.	6–12 months	Cure 10/10 (100)	[90]
Brazil	Open-label	10	19.0	Amphotericin B cholesterol dispersion	2.0mg/kg/d for 7 d. I.V.	6–12 months	Cure 10/10 (100)	[90]
Brazil	Open-label	10	16.5	Amphotericin B cholesterol dispersion	2.0mg/kg/d for 5 d. I.V.	12 months	Cure 9/10 (90)	[91]
							Relapse 1/10 (10)	
Brazil	Open-label Phase II	13	7.6	Liposomal amphotericin B	14mg/kg (total) . I.V.	6 months	Cure 8/13 (61)	[86]
							Failure 1/13 (8)	
							Relapse 4/13 (31)	
Brazil	Open-label Phase II	4	7.5	Liposomal amphotericin B	10mg/kg (total) I.V.	6 months	Cure 4/4 (100)	[86]
Brazil	Open-label Phase II	15	10.1	Liposomal amphotericin B	20mg/kg (total) I.V.	6 months	Cure 13/15 (87)	[86]
							Relapse 2/15 (13)	
Brazil	Open-label, dose-escalating trial	4	19.0	WR6026 (sitamaquine)	1.0mg/kg/d for 28 d. Oral.	12 months	Cure 0/4 (0)	[89]
Brazil	Open-label, dose-escalating trial	6	32.8	WR6026 (sitamaquine)	1.5mg/kg/d for 28 d. Oral	12 months	Cure 1/6 (17)	[89]
Brazil	Open-label, dose-escalating trial	6	23.8	WR6026 (sitamaquine)	2.0mg/kg/d for 28 d. Oral.	12 months	Cure 4/6 (67)	[89]
Brazil	Open-label, dose-escalating trial	5	23.8	WR6026 (sitamaquine)	2.5mg/kg/d for 28 d. Oral	12 months	Cure 1/5 (20)	[89]
Brazil	Open-label, dose-escalating trial	1	22.0	WR6026 (sitamaquine)	3.25mg/kg/d for 28 d. Oral	12 months	Cure 0/1 (0)	[89]

Dietze et al (1993) reported an open-label dose-escalating trial with amphotericin B colloidal dispersion (Amphocil) in two small groups of patients who showed similar cure rate suggesting that the 7 days was as effective as the 10 days regimen [90]. In 1995 the same authors reported another open-label trial with Amphocil with a shorter regime of 5 days, observing an episode of relapse [91]. Berman et al (1998) reported the results of an open-label phase II trial with three therapeutic regimens consisting of liposomal amphotericin B 10, 14 or 20 mg/kg total dose; the reported outcomes were cure, failure and relapse and the follow-up period was of six months. This paper suggested that the lower 10mg/kg total dose was less efficacious than the higher 20mg/kg total dose [86]. Dietze et al (2001) concluded from an open-label dose-escalating safety and efficacy trial that sitamaquine was not efficacious for the treatment of VL in young adults. Severe adverse events described as renal toxicity lead to trial interruption when using the higher dose of 3.25mg/kg/d [89].

#### I: Key questions for control

1. What is the current standard of care for VL treatment in the Americas?
2. What is the case for combination therapy for VL in the Americas?
3. What is the standard of care in VL/HIV co-infection?

#### II: Questions for research

1. What is the current efficacy of pentavalent antimonials, amphotericin B deoxycholate and the liposomal formulations, miltefosine and drug combinations for VL treatment in the Americas?
2. Are there more efficacious, safer, and simpler therapeutic schemes for VL than the current ones?
3. Can a clinical prognostic score for treatment failure be developed to identify those cases most in need for intensive care?
4. What is the role of non-parasite targeted drugs such as immunomodulators, antibiotics and others in VL treatment?

### Vector and animal reservoir control

Incidence and prevalence estimates of canine VL in the Americas have been reported from several foci [2], [40], [92]–[95], but the specific relationship between canine and human VL cases is not well understood. Transmission in the dog population is mainly due to infected sandfly bites but alternative routes have been proposed such as sexual transmission and other potential insect vectors [96]–[98]. The control of the animal reservoir is complex and frequently involves combined interventions. The Brazilian Control Program recommends a strategy based on canine culling and vector control with insecticide spraying. Insecticide-impregnated collars for dogs and canine vaccination are not currently recommended as public health control measures [99].

**Figure 4:**
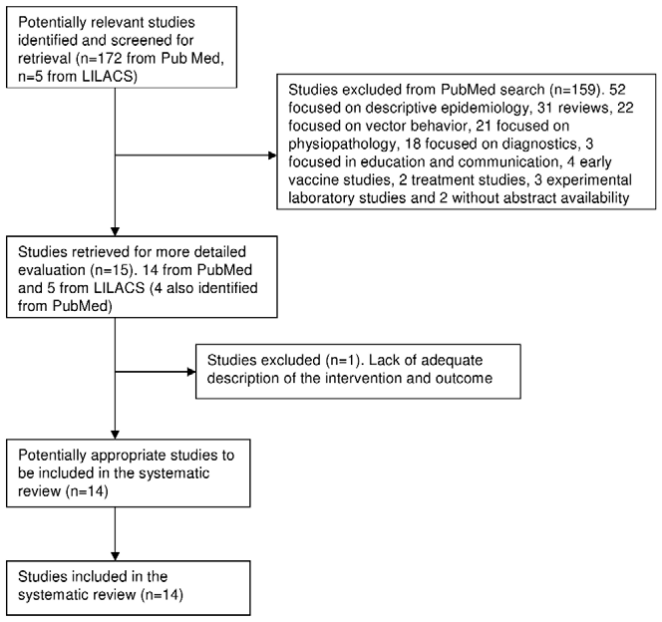
Flow of inclusion of studies on VL control.

One-hundred seventy-two papers were retrieved from Medline/PubMed using the search strategy cited above. The LILACS search was performed using the term **visceral leishmaniasis** because no document was retrieved when using the PubMed approach. The LILACS search was less specific and 519 documents were retrieved; 514 documents comprised an extensive spectrum of research irrelevant for the purpose of this paper and four of the five relevant papers were already identified through the PubMed search. After reading the titles and the abstracts and hand searching reference lists for related papers, fourteen were selected for full text reading because the main subject was at least one intervention for control VL (Figure 4) [50], [55], [100]–[111].

Magalhães et al (1980) published a retrospective –non controlled- study on the impact of a combined intervention consisting of human VL case treatment, culling of seropositive dogs and insecticide spraying with DDT in 19 municipalities of the Rio Doce Valley, State of Minas Gerais, Brazil reporting the disappearance of human symptomatic cases after 15 years of application of this strategy [100].

Dietze et al (1997) reported a field trial of dog screening and culling, based on twice-yearly screening with DOT-ELISA. This trial was conducted in three rural valleys, State of Espirito Santo, Brazil, two benefiting from the intervention and one used as control. At 6-months there was a 16% reduction of seroconversion rate in dogs (36% in the intervention vs. 52% in the control group), but this difference was not significant [101].

Braga et al (1998) reported the comparison of two strategies of dog screening-and-culling: screening by ELISA was compared to IFAT as routinely recommended by the National Control Program. The main difference consisted in the lag times after blood sampling (7 days for ELISA vs. 80 days for IFAT). The trial was conducted in a rural area of Northeastern Brazil where 28 communities were systematically allocated to one of the two groups. In the ELISA arm, reduction of canine seroprevalence was higher, probably due faster dog removal plus higher sensitivity of the ELISA test [102].

Ashford et al (1998) reported a controlled intervention trial of seropositive dog removal in an endemic area of the State of Bahia, Brazil. The intervention area was subjected to screening with FAST-ELISA and removal of seropositive dogs, in the control area no intervention was carried out. A significant reduction of dog seroconversion rate in the intervention area as compared to control was observed, and a significantly lower number of VL cases reported to health facilities in the intervention area [50].

Paranhos-Silva et al (1998) report a follow- up study of several clusters of seronegative dogs in Jequié, State of Bahia, Brazil. The initial prevalence of infection among 1681 dogs was 23.5%. After serological screening every six months for 18 months and removal of the seroconverters, the annual incidence rate of infection was 6.55 cases/100dog-years. The migration of dogs between clusters was 2.3 cases/100 dog-years. This study is relevant because as highlights the challenges posed by dog migration for any control program dealing with the canine reservoir [103].

Da Silva et al (2000) reported a phase III vaccine field trial in seronegative dogs screened with IFAT and FML-ELISA and exposed to fucose-mannose-ligand vaccine in three subcutaneous doses at 21 day intervals. Control arm was treated with saline placebo. Endpoints were symptomatic VL or death, seroconversion rates in FML-ELISA and conversion of leishmanin skin test composed of crude *L. donovani* antigen. Follow-up evaluations were performed at 2, 7, 13 and 24 months. A significant difference in the three endpoints was observed during the trial. The overall efficacy to prevent symptomatic VL disease was 75% [104].

Giffoni et al (2002) reported the effect of application of a 65% permethrin spot-on formulation on canine VL infection and sandfly abundance. A decrease of canine VL prevalence was observed in the intervention area compared with increased prevalence in the control area. No effect was observed on sandfly population [105].

Feliciangeli et al (2003) described a controlled trial of pyrethroid (λ-cyhalothrin) indoor spraying every 5 month and organophosphate (fenitrothion) ultra-low volume spatial fogging around the houses twice a month for ten months in one intervention compared to one control area. The main vector captured was *Lu. longipalpis*. A significant decrease of sandfly abundance was observed, with a residual effect of indoor spraying of 3 months. Main limitation of this study was the specific construction style of the houses: completely cemented, plastered and oil-painted walls and zinc roofs, which lowers its external validity [106].

De Oliveira et al (2003) reported the evaluation of routine combined control measures of seropositive dog-culling and insecticide spraying during six years. The intensity of the application of control measures correlated with human VL incidence, the coverage of canine surveys, the number of canine surveyed and the number of buildings submitted to insecticide spraying [107].

Reithinger et al (2004) reported a controlled field trial to evaluate the effectiveness of insecticide impregnated collars to prevent infection detected through serological tests or DNA detection by PCR assay in one intervention compared to one control area. The authors failed to detect a significant difference between groups in the incidence of new infections but they demonstrated a significant reduction of antibody titers in the collar protected dogs. Mathematical modeling using the results obtained in this study suggests that dog collars would be a better alternative than dog culling [55].

Moreira et al (2004) reported the incidence rates of canine *Leishmania* infection in a cohort of dogs submitted to an optimized culling strategy consisting of: (i) ELISA screening of serum samples; (ii) shortening of the time interval from serodiagnosis to removal of dogs; (iii) screening a high proportion of the dog population. They demonstrated that the incidence of canine infection remained stable through 2.5 years of observation under this strategy but the study had no control arm for comparison. A high replacement rate by susceptible puppies and already infected dogs was observed [108].

Courtenay et al (2007) reported the barrier effect, the 24-h mortality rate and the human landing rates of *Lu. longipalpis* in households using deltamethrin-impregnated bednets compared others using untreated bed nets. The study described a 39% increase in barrier capacity of the impregnated bednets, 80% reduction in sandfly landing rates on humans and 98% increase in the 24-h sandfly mortality rates. The study was done under field conditions with a small number of observations during a very short period of exposure to the treated bednets (three days) and the residual effect was not measured. However this intervention should be explored further because it could bring an additional benefit in areas where malaria is also endemic [109].

Costa et al (2007) reported a randomized community intervention trial to compare the effect of four strategies on human VL, as follows: (i) spraying houses and animal pens with pyrethroid insecticide; (ii) spraying houses and eliminating seropositive dogs; (iii) combination of spraying houses and animal pens plus eliminating seropositive dogs; and (iv) spraying houses only as the reference comparator. The outcome was evaluated by measuring incidence of seroconversion in humans six months after the application of interventions. The results indicated a positive effect of canine removal on incidence of leishmanial infection in men but surprisingly, the combination of dog culling plus outdoor spraying of peridomestic animal shelters failed to demonstrate any effect. The relevance of this study is that it constitutes the first attempt to measure the effect of combined interventions on human VL incidence [110].

De Souza et al (2008) reported a randomized community intervention trial to compare the effect of (i) pyrethroid insecticide spraying; (ii) pyrethroid insecticide spraying plus culling of seropositive dogs with (iii) no intervention. The interventions were maintained for two years and outcomes were registered every year, insecticide spraying was performed every 6 months. Although a lower incidence was observed in the groups submitted to interventions and that reduction was more intense after two years, the study failed to detect statistically significant differences [111].

The summarized characteristics and main limitations of these studies are shown in Table 4.

**Table 4:** Main characteristics of selected studies reporting effects of control interventions against visceral leishmaniasis in Latin America.

Country and period	Study setting	Intervention	Comparison	Number of subjects in intervention and control arm	Follow-up	Outcomes (measures)	Effect measures	Results	Main limitations	Ref.
**CULLING**										
Brazil Period: NR	Three adjacent rural valleys in the Espírito Santo State	Culling of seropositive dogs 0 and 6 months after inclusion	2 intervention valleys vs 1 control valley	Intervention valleys – 267 humans	12 months	Human infection (seroconversion in Dot-ELISA)	Difference in infection rates in humans and dogs in intervention vs control valleys	0% difference in human seroconversion rates	i) non-randomized	[101]
				Control valley – 202 humans		Canine infection (seroconversion in Dot-ELISA)		Not significant difference (4%) in dog seroconversion rate	ii)low number of clusters for comparison (2∶1)	
				Dogs – NR					iii) 26.5% loss to follow-up in humans	
									iv)small sample of domestic dogs,	
									v)Canine loss to follow-up not described	
Brazil Period: NR	28 rural villages in the São Luiz do Curu Municipality in the State of Ceará	Rapid culling based on ELISA *versus* conventional culling based on IFAT	1 intervention group vs 1 control group composed	Intervention group – 276 dogs	10 months	Canine infection (seroconversion in ELISA)	Difference of seroprevalence between groups	Significant reduction of seroprevalence in the intervention group (27% *versus* 9%; P = 0.001)	i) baseline seroprevalences significantly different	[102]
				Control group – 254 dogs					ii) impossibility to disentangle the effect of the time to dog removal from the effect of the lower sensitivity of the IFAT test	
Brazil 1989–1993	Two neighborhoods of the city of Jequié in the Bahia State	Yearly culling of seropositive dogs	1 intervention area vs 1 control	Initial number of dogs in the intervention area – 235	5 years	Canine infection (seroconversion in FAST-ELISA)	Difference in cumulative incidence of canine infection between neighborhoods	Canine infection cumulative incidence did not change (P = 0.07)	i)small sample size	[50]
				Dogs in the control area – NR		Human pediatric VL cases	Difference in incidence of pediatric VL	Pediatric VL incidence decreased in the intervention area (P<0.01)	ii)ineffective dog removal	
				Humans – NR					iii) differential losses during follow-up	
									iv) low human VL incidence	
									v) non-randomized	
									vi) areas submitted to heterogeneous follow-up	
Brazil Period: NR	Urban and periurban areas of the Jequié Municipality in the Bahia State	Culling of seropositive dogs at baseline and every 6 months	Before/after	Cohort of 1286 susceptible dogs, no controls	18 month	Dog emigration Canine infection (seroconversion in ELISA) evaluated every 6 months	Dog emigration rate and canine infection incidence	Emigration rate: 2.26 cases/100 dogs-year	Intervention of dog culling was not directly evaluated	[103]
								Overall annual incidence of 6.55 cases/100dogs-year		
								Two risk strata for seroconversion rates with higher risk in the periurban versus downtown clusters		
Brazil 1997–2000	Jequié city, State of Bahia	Culling of seropositve dogs at baseline and every 8 months	Before/after	Dynamic cohort of 447 dogs at study entry	31 months	Canine infection (seroconversion in ELISA) evaluated every 8 months	Difference in incidence rates every 8 months	No significant changes in the incidence rates through the study period	i) no control arm	[108]
**INSECTICIDE MATERIALS**										
Venezuela 1999	Two rural villages in the Island of Margarita	Pyrethroid λ-cyhalothrin sprayed indoors every 5 months; and organophosphate fenitrothion through peri-domestic fogging 16 times during the year vs control( no intervention)	1 intervention village vs 1 control	Five houses in each village (control and intervention)	12 months	Plebotomine sandfly density	Differences in indoor and outdoor sandfly density between intervention and control groups	Significant reduction of the sandfly density in the intervention village (P<0.001)	i) small sample size	[106]
									ii) low external validity	
Brazil 1999	Two localities in the Corumba municipality, State of Mato Grosso	65% permethrin spot-on three times monthly	1 intervention locality vs 1 control	Intervention area: 150 dogs	5 months	Canine seroconversion in IFAT	VL prevalence three months after treatment	Reduction of VL prevalence in the intervention area (19.3% to 10.8%)	i) non-comparable baseline prevalence	[105]
				Control area: 146 dogs				Increase of VL prevalence in the control area (4.1% to 16.8%)	ii) low sensitivity of the test used to define infection (IFAT)	
									iii) significant losses during follow-up	
Brazil 1999–2000	Two neigborhoods in the Capitão Eneas Municipality, State of Minas Gerais	Deltamethrin-impregnated dog collars vs none intervention	1 intervention vs 1 control area	Intervention area: 251 dogs	5 months	Canine infection (conversion in ELISA or peripheral blood PCR-hibridisation assay)	Difference in the infection rates between groups	11.9% intervention group vs 17.6% in the control group (P = 0.24)	i) the one to one comparison,	[55]
				Control area: 190 dogs					ii) non comparable baseline prevalence of VL infection between groups	
									iii) high rate of loss of follow-up,	
									iv) high frequency of collar loss and migration of dogs	
Brazil 2003	Salvaterra municipality in the Marajó Island, State of Pará	Deltamethrin impregnated bednets vs untreated bednets	Crossover study	Two houses in each group	Three consecutive nights	Bednet barrier effect, human landing rates and 24h sandfly mortality rates	Differences in barrier effect magnitude, landing rates and sandfly mortality rates	39% increasing in barrier effect	i) small number of observations	[109]
								80% reduction in human landing rates	ii) short exposure period	
								98% increasing in sandfly mortality		
**COMBINED INTERVENTIONS**										
Brazil 1965–1979	Mainly rural communities of 19 Municipalities of Minas Gerais State	Dog culling + human treatment + DDT spraying of houses	Before/after	81,162 dogs, unreported number of human subjects and no control	15 years	Human VL (clinical AND/OR positive CFR AND/OR positive parasitology)	Incidence of human VL before/after	Human VL disappearing ∼0%	i) no controls	[100]
						Canine VL (seroconversion in CFR)	Incidence of canine VL before/after	Canine VL ∼0%	ii) intensity and periodicity of intervention poorly described	
									iii)low sensitivity of the complement fixation test to detect canine infection	
									iv) passive reporting cases as the data source for endpoint in humans	
									iv) the human and canine population exposed to the control measures was not reported	
Brazil 1995–1996	One neighborhood of the city of Teresina, State of Piaui	(A)spraying houses and animal pens with insecticide	Random allocation of 34 clusters to one of four arms	213 susceptible humans (120 evaluated, numbers of susceptible humans in each intervention were not reported)	12 month	Human infection (seroconversion in ELISA) at least 6 months after intervention	Difference in incidence rate	Significant reduction in incidence in the group exposed to intervention B	i) non comparable baseline VL incidence between the house spraying group and the other three groups	[110]
		(B) spraying houses and infected dog-culling		Control arm: group submitted to house spraying (D)				No significant decrease in incidence in A and C intervention groups	ii) high percentage of loss to follow-up of susceptible individuals (44%),	
		(C) combination of (A) and (B)							iii) the suboptimal sensitivity and specificity of the method to measure seroconversion (crude antigen-ELISA)	
		(D) spraying houses								
Brazil 1995–2000	Municipality of Feira de Santana, State of Bahia	Culling of seropositive dogs and house and animal shelters pyrethroid insecticide spraying	None	124 localities (30 urban and 58 rural with human VL incident cases and 36 localities around them)	6 years	Human VL incidence	Correlation between measure coverage and frequency with human VL incidence	Positive correlation with number canine surveys, coverage of canine surveys and number of sprayed buildings	i) secondary source data	[107]
									ii) lack of a control arm	
Brazil 2004–2006	Two neighborhoods of the Feira de Santana city, State of Bahia	(A) No intervention	Intervention was randomly allocated to one of 3 areas in each neighborhood	Dynamic cohort of 2362 children (688, 782 and 892 allocated to interventions A, B and C, respectively	27 months	Human incidence (seroconversion in ELISA)	Relative risk for infection every 12 months	Lower but not significant incidence decrease in the intervention areas	i) low study power,	[111]
		(B) insecticide spraying							ii) significant losses during follow-up	
		(C) combination of insecticide spraying and seropositive dog culling								
**DOG VACCINE**										
Brazil Period: NR	São Gonçalo do Amaranto Municipality in the Rio Grande do Norte State	Vaccination with Fucose-Mannose-ligand antigen, 3 subcutaneous doses at 21 day intervals	Intervention arm – FML vaccine	Intervention – 58 seronegative healthy dogs (in IFAT and FML –ELISA)	24 months	Symptomatic VL at 2, 7, 13 and 24 months	Difference in symptomatic VL rate (cumulative at 24 months follow-up)	8% (intervention) vs 67% (placebo) symptomatic VL	i) impossibility of accurate evaluation of the infection rate because the vaccine product and probably the repeated leishmanin doses interfered with the serological response with more than half of control subjects showing positive FML-ELISA tests,	[104]
			Control arm –Saline placebo	Control – 59 seronegative dogs (in IFAT and FML-ELISA)		FML-ELISA seroconversion at 2, 7, 13 and 24 months	Differences in seroconversion rates (cumulative at 24 months follow-up)	100% (intervention) vs 68% (placebo) seroconversion rate	ii) no random allocation	
						Leishmanin conversion (*L. donovani* antigen) at 2, 7, 13 and 24 months	Difference in leishmanin positive rate (cumulative at 24 months follow-up)	94% (intervention) vs 14% (placebo) leishmanin positive rate	iii) lack of baseline data on dog characteristics	

CFR = complement fixation reaction. NR = Not reported.

#### Key questions for control

1. What is the most cost-effective control strategy for VL?
2. How to conduct a valid impact evaluation?
3. Can general support measures (nutritional rehabilitation and housing improvement) be targeted to VL endemic areas?
4. What is the potential impact of current dog vaccines on transmission?

#### Questions for research

1. What are the determinants of dog infectiousness for the sandfly vector?
2. What are the determinants of dog susceptibility to infection?
3. What is the efficacy of current dog vaccines to prevent disease in dogs and to reduce infectiousness for the sandfly vectors?
4. What is the effectiveness of insecticide- impregnated dog collars to prevent human and canine infection?
5. What is the efficacy/effectiveness of alternative vector control devices (insecticide treated nets, curtains, etc) in the prevention of VL?

## Discussion

### Research gaps

This review of evidence related to VL control in Latin America revealed that a lot of work remains to be done in order to clarify the dynamics of *Leishmania* transmission in human, canine and vector populations. The exact burden of disease remains largely unknown. The increasing trend of VL cases observed in Brazil and the spread of transmission to previously not affected areas raise doubts about the impact of ongoing control measures. The determinants of human infection and of symptomatic disease are also poorly understood with the exception of the nutritional status in young children.

To diagnose VL in humans the rK39-ICT has clear advantages over the IFAT or ELISA based tests that are widely used in Latin America. The DAT assay has shown similar diagnostic performance but is not as user-friendly as the rK39. The research priorities in this field should be geared towards diagnostic accuracy studies in large prospective trials (phase-3) and to study diagnostic performance in specific groups such as HIV co-infected patients. Current treatment practice in VL in Latin-America is based on rather weak scientific evidence. It is worrisome that case fatality rates remain high and are even increasing, at least in Brazil. The lack of clinical evidence from the region is very worrying. We retrieved not a single phase-3 randomized controlled trial on VL conducted in the Americas. Nowadays, one phase-2 trial with miltefosine is ongoing and two Brazilian large randomized controlled trials with liposomal amphotericin B, amphotericin B deoxycholate and meglumine antimoniate are expected to initiate recruitment in 2009. The research priorities include well-designed clinical trials with pentavalent antimonials, amphotericin B deoxycholate and the liposomal formulations, miltefosine and drug combinations. Although the resistance to antimonials observed in India is less relevant in Latin America, drug combinations are attractive because their potential for shortening treatment schemes and reduction of toxicity. Clinical factors associated with treatment failure should be studied to contribute to the development of a prognostic score that allows early interventions to reduce case fatality rates [14],[87].

Control interventions targeting the dog reservoir for culling/treatment require accurate assays able to detect the asymptomatic infections as well as the symptomatic dogs. Validating such tests is no easy task, as there is no adequate gold-standard for the diagnosis of asymptomatic infection. PCR-assays seem to be very attractive but estimating their accuracy and reproducibility still constitutes a research priority. Moreover, novel screening strategies based on combined, parallel or sequential use of current available tests needs to be validated. Another challenge faced in canine diagnosis is the distinction of positive serology results produced by natural infection from those induced by vaccines. The development and proper validation of tests with capacity to discriminate both phenomena are crucial to avoid interference with concomitant interventions including dog culling and vaccination in the same area. Furthermore, the study of the determinants of dog infectiousness for the sandfly vector is essential to define the best culling strategy [112],[113] and the determinants of dog susceptibility to infection [114] is crucial for the design of canine vaccine trials.

Some of the problems with the design of the community intervention trials we reviewed are related to the lack of accurate diagnostic methods to define the relevant outcomes in the human and canine population. Furthermore, the definition of a control group is challenging because of an obvious ethical dilemma. The heterogeneity of disease transmission within the study area often generated imbalances in the baseline comparisons among groups and the random allocation process is also complex because of the mobility of the human, canine and vector population. Most of the reported community trials used a too limited number of clusters for comparison (usually a one to one comparison). In spite of all those limitations a relevant number of reports could be reviewed in detail, showing no strong evidence for a significant impact on VL transmission for any of the interventions reviewed. Canine culling seems to be the least acceptable intervention at community level for obvious reasons and has low efficiency due to high replacement rate of eliminated dogs with susceptible puppies [103],[115],[116]. Vector control interventions are better accepted by the affected populations and mathematical models suggested encouraging efficacy, but they need further study. Better knowledge of vector seasonality and behavior is required for proper timing of these interventions. The current evidence indicates that spatial fogging is useless and that the residual effect of house wall spraying is very short [106],[117]. Insecticide impregnated collars seem to have a longer residual effect [56] and theoretical advantages over the other methods and should be studied in larger and well-designed controlled trials. The potential emergence of resistance to insecticides should also be considered for the long-term planning of any vector control intervention [118].

Canine and human vaccine development needs to be prioritized. The dog vaccines already registered in Brazil have some protective effect against canine VL but none of them were properly evaluated as control measures against human VL [119],[120]. Such evaluation is challenging as field trials should include relevant canine endpoints, related to dog infectiousness for the sandfly vector, as well as relevant human endpoints, that include symptomatic and asymptomatic infections in order to obtain precise estimates of the vaccine effect on transmission rates. Human vaccine development is expected to take at least several years to obtain efficacious and safe candidates for clinical trials. Furthermore, the surrogate markers of the desired protective effect are not well understood and the definition of target population for such products will be a matter of intense debate.

The role of sylvatic and peridomestic animals such as foxes, marsupials and rodents in some relevant VL transmission scenarios deserves more specific research [6].

Last but not least, in countries such as Brazil, where the government has put the elimination of hunger as a political priority, targeted nutritional support in VL risk areas would be an interesting and probably cost-effective intervention from a societal perspective. Similarly, schemes for the improving of housing and waste management as well as other general measures involving active community participation should be encouraged [121],[122].

Finally, the strengthening of the surveillance system capacity is essential to avoid the underreporting of human cases [123] and to follow-up the infection behavior in canine population. Strong surveillance will certainly contribute to improve data quality for decision-makers in this complex scenario.

### Concluding remarks

The elimination of zoonotic VL in Latin America is not (yet) a realistic goal taking into consideration the complexity and diversity of its transmission scenarios, the scientific knowledge gaps and the lack of adequate and properly validated interventions. Many countries perceive the burden of leishmaniasis as negligible; there is not much political support nor funding for VL control. The zoonotic nature of transmission is an additional constraint that limits the impact of the few known effective prevention and control interventions. Nonetheless we believe the improved control of VL is possible if the region builds the political will, develops a more coherent regional control policy, and invests in better case management and epidemiological surveillance systems. The implementation of a focused research agenda to support such control initiative is essential.

## Supporting Information

Checklist S1 PRISMA checklist.
(0.07 MB DOC)
